# Bilateral paediatric cataract surgery - outcomes of 298 children from Kinshasa, the Democratic Republic of the Congo

**DOI:** 10.4314/ahs.v20i4.36

**Published:** 2020-12

**Authors:** Janvier Kilangalanga Ngoy, Thomas Stahnke, Serge Dinkulu, Emile Makwanga, Astrid Moanda, Georgette Ngweme, Edith Mukwanseke, Günther Kundt, Frank Thiesen, Adrian Hopkins, Rudolf F Guthoff

**Affiliations:** 1 Eye Department, St Joseph Hospital/CFOAC, Kinshasa, DRC; 2 Rostock University Medical Center, Department of Ophthalmology, Rostock, Germany; 3 Programme National de Sante Oculaire et Vision (PNSOV), Kinshasa, DRC; 4 Réhabilitation à Assise Communautaire (RAC/CBR), Kinshasa, DRC; 5 Rostock University Medical Center, Institute for Biostatistics and Informatics in Medicine and Ageing Research, Rostock, Germany

**Keywords:** Paediatric cataract surgery, bilateral cataract, outcomes

## Abstract

**Introduction:**

The leading cause of childhood blindness globally is paediatric cataract. Bilateral cataract surgery can help to improve visual performance and to diminish the burden of childhood blindness.

**Objective:**

To report in a retrospective observational cohort study the long-term outcomes of 298 children who had bilateral cataract surgery with IOL implantation from 2001–2016 in Kinshasa.

**Methods:**

A standardized surgical treatment of paediatric cataract was practiced on 298 children. Patient's follow-up, complications, and visual outcomes were recorded and analysed.

**Results:**

The mean age was 5.7 ± 4.3 years and males were predominant (64.9%). Most of children were living mainly in urban poorest areas (96.3%). Strabismus, nystagmus and microcornea were encountered in 20.1%, 25.1% and 8.7% of children, respectively. Using WHO criteria most of patients were classified as blind preoperatively and 81.9% of them had improved visual outcomes after surgery. Main reasons for reduced vision during follow-up were secondary cataract (5.7%), IOL decentration (1.2%), retinal detachment (1.2%), and secondary glaucoma (1.5%).

**Conclusion:**

In spite of the post conflict challenges, elimination of cataract blindness in children remains a priority. Children present at a late age for surgery and long term follow-up is poor. There is need for program strengthening in these areas.

## Introduction

Cataract is the main cause of blindness among children in Africa, replacing vitamin A deficiency and measles thanks to successful immunization programs 1. Successful long-term outcomes of paediatric cataract management require high quality surgery and long-term follow-up both to manage complications and to reach an optimal visual outcome. Blindness in children with cataract is not only attributed to the cataract itself but also to deprivation from early onset and delayed presentation, resulting in delayed surgery, surgical complications, or associated ocular abnormalities 2–4. Challenges to improve outcomes in limited resources countries include the following:

1. children often present late after years of deprivation
2. parents may not be able to afford surgery, optical and low vision correction as well as follow-up costs
3. children often do not return for follow-up.

In Kinshasa, a survey carried-out in blind schools found paediatric cataract to be the main cause of childhood blindness. Out of 139 blind, 47 suffered from undiagnosed bilateral cataracts (33.7%) 5. To address this issue, Saint Joseph's Hospital launched a paediatric eye care unit in collaboration with the University of Rostoc (Germany) in 2001.

A church-based volunteer program was used to identify children with severe visual impairment or blindness in 31 out of 165 parishes in the Archdioceses of Kinshasa. This concept is described in detail elsewhere (Role of a community-based program for identification and referral of paediatric cataract patient in Kinshasa, Democratic Republic of the Congo) 6. St. Joseph's Hospital developed into a referral centre for the region performing high quality cataract surgery with bilateral lens implantation. The associated Community Based Rehabilitation Program (CBR) took the main burden for follow-up, patching, social and educational rehabilitation and low vision therapy. Most of the costs for surgery, hospitalization and working with families to encourage integration into the educational system were sponsored by various international funds and the Christian Blind Mission (CBM).

The aim of this study was to analyse retrospectively the visual outcome and to lay foundations for a refined management for the future. A prospective study sponsored by the «Else Kröner Fresenius Stiftung» (Bad Homburg, Germany) is being developed.

## Methods and Patients

**Figure 1:**
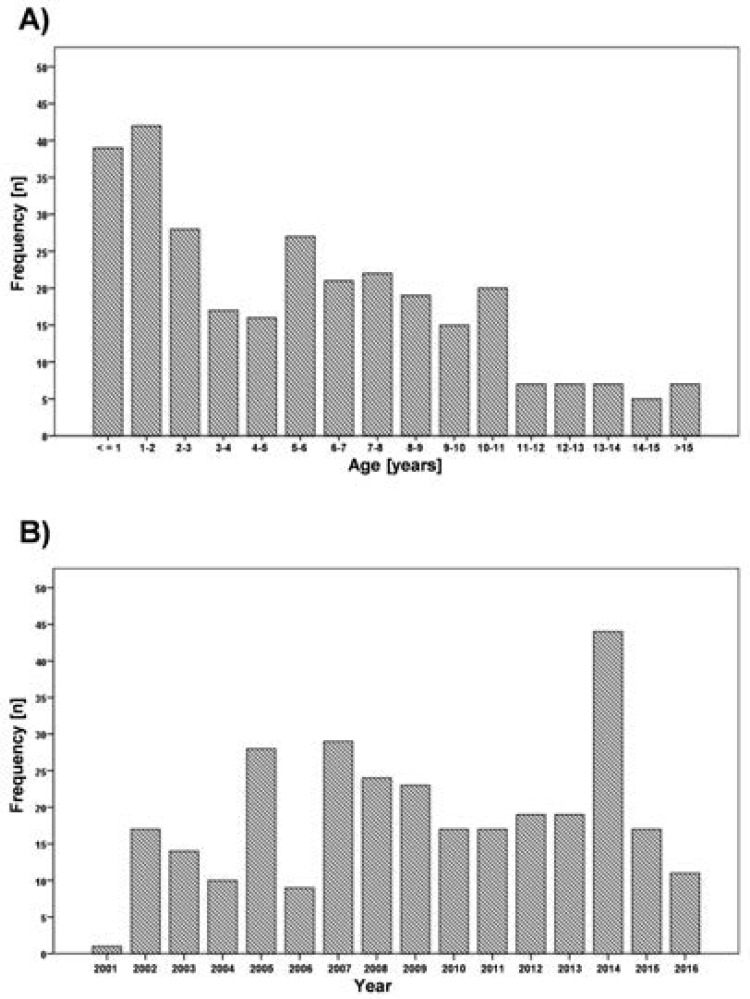
Age distribution of 298 children with bilateral cataract (A) and number of surgeries per year (B).

Children aged 0.4 to 17 years (Fig. 1 A) with bilateral cataract, undergoing surgery between 2001 and 2016 (Fig. 1 B) were included in this study. The most common type of cataract was total lens opacity in 424 eyes (71.1%). They were selected out of a total of 11,106 children having severe visual impairment or blindness identified by the volunteer community workers in the CBR program of the Archdiocese Kinshasa 6. 254 children (85.2%) in the study were recruited by cataract finders working for the CBR program during identification sessions in parishes. They were referred to the hospital by the volunteers. Surgical costs were subsidised by the CBR program through charitable donations. Almost all children (n=287, 96.3%) were geographically from the urban poor areas with very limited family income. Overall 298 children with bilateral cataract were included and patient paper records were analysed via an electronic data base using a remote program with a server located at the Institute for Biostatistics and Informatics in Medicine and Ageing Research, Rostock University Medical Center, Germany.

All children were hospitalized together with at least one family member for a time between 4 and 7 days. Surgery was performed in all children under general anaesthesia. Ultrasound biometry (A-SCAN Biometer AL-100, Tomey GmbH, Nuremberg, Germany) and keratometry (KM-500 PR1, NIDEK CO., LTD., Gamagōri, Japan) took place during this procedure. For the IOL power calculation the SRK-T formula was used if axial length (AL) was > 26 mm. Hoffer Q formula was used for all eyes with an AL < 22 mm and Holladay 1 formula for eyes with AL between 22 – 26 mm. To avoid higher myopic shifts during adolescence, IOL power was chosen to give approximately 20.0% under correction in children younger than 2 years and 10.0% for those between 2 to 8 years according to the Dahan et al. protocol 7. Emmetropia was the refraction target for children 8 years plus.

Surgery was performed under general anaesthesia using isoflurane and propofol. Minimally invasive cataract surgery techniques were used: following capsulorhexis, in most children manual irrigation/aspiration technique was sufficient to remove all lens materials, harder nuclear elements were delivered through a tunnel incision at a 12 o'clock position. Routine posterior capsulorhexis or capsulotomy was performed combined with an anterior vitrectomy using the Vitron unit (Geuder AG, Heidelberg, Germany). The intraocular lenses were inserted through a tunnel incision.

In nearly all cases Polymethylmethacrylat (PMMA) lenses with an optic diameter of 5 or 5.5 mm and 12 mm overall diameter were inserted into the capsular bag, and if not in the ciliary sulcus. Although we know that PMMA lenses can lead to complications such as inflammation or secondary cataract, we decided to use this type of lens in view of the given poor socio-economic conditions for reasons of availability and affordability. After removal of viscoelastic material, the water-tightness of the incisions was checked and if necessary 10-0 nylon sutures were applied. Sutures remained in place and were only removed in cases where irritations occurred.

Bilateral simultaneous surgery was carried out on children when the anaesthesia risk was found to be too high for further exposing the child to a second procedure (Tab. 1).

**Table 1:** Number of eyes operated unilaterally or bilaterally and reimbursement of surgery costs

Surgery and financing	Frequency [n]	Proportion [%]
Unilateral surgery	(52 patients) 104	(17.5) 18.2
Bilateral surgery, both eyes simultaneously	(234 patients) 468	(78.5) 81.8
CBR program reimbursement	470	82.3
Partly CBR program, partly relatives and friends	81	14.1
Parents	21	3.6

Post-operatively all children got at least five times local steroids (Prednisolone) and three times local antibiotics (Gentamicin) per day. When needed the treatment was extended to hourly application of steroids for 2–3 days. Refraction was carried out at the Eye Department during follow-up visits, and glasses were distributed by the CBR program. For visual acuity (VA) measurements a variety of charts was used: Lea symbols matching tests and Snellen charts. VA was categorized according to the World Health Organization classification (not impaired: ≥ 6/18, visual impairment: 6/18 to 6/60, severe visual impairment: 6/60 to 3/60, and blind: < 3/60). Routine follow-up visits were scheduled after 3 weeks, 3 months, and 1 year with further periodical review, as required. For statistical evaluation, the term last visit was also included for final analysis.

The study complied with local laws and the principles of the Declaration of Helsinki. When additional procedures were suggested, parents were informed and their consent was required. A consent form was presented, explained to parents and their signature was required.

## Results

Primary posterior capsulotomy and anterior vitrectomy were performed in 540 eyes (90.6%). 24 eyes with severe microcornea or with lens subluxation were left aphakic. IOL's were placed in the bag in 568 eyes, in 3 eyes in the ciliary sulcus, and 1 iris supported lens was used.

Due to an intensive post-operative steroid regimen, no cases of intraocular inflammation were observed during follow-up examinations. Post-operative complications include secondary cataracts (15 patients, 5.8%), retinal detachment (3 patients, 1.2%), severe IOL decentration (3 patients, 1.2%), and secondary glaucoma (4 patient, 1.5%). Hyphema was found in 4 patients early after surgery and all resolved within 1 week. A transient corneal edema was found in 1 eye (Tab. 2).

**Table 2:** Distribution of postoperative complications

Postoperative complications	Frequency [n]	Proportion [%]
Hyphema (resolved within 1 week)	4	1.5
Corneal edema (transient)	1	0.4
Secondary cataract	15	5.8
Retinal detachment	3	1.2
Severe IOL decentration	3	1.2
Secondary glaucoma	4	1.5

The mean post-operative intraocular pressure (IOP) was 18 ± 8 mmHg (min. 6 mmHg – max. 56 mmHg). One child developed phtisis bulbi after cyclophotocoagulation for persistent high intraocular pressure. Eleven children underwent Yag laser capsulotomy for secondary opacification of the visual axis and another 4 children, unable to cooperate for YAG-laser capsulotomy, got a surgical removal of lens opacities.

Glasses were prescribed after refraction with a mean of + 0.5 ± 1.5 diopters (min. - 9 diopters, max. + 5 diopters). Patching was mainly performed by volunteers at the child's home 6 days a week and by parents for a number of children. Ten children received vision aids from the low vision unit at the CBR program (loupes and telescopes). Optical assessment by retinoscopy was possible in 556 eyes (93.3%).

A total of 290 follow-up visits are included in the data base. The mean number of follow-up visits per patient was 4.22 ± 2.81 visits with a maximum of 17 visits (median 3 visits).

**Figure 2:**
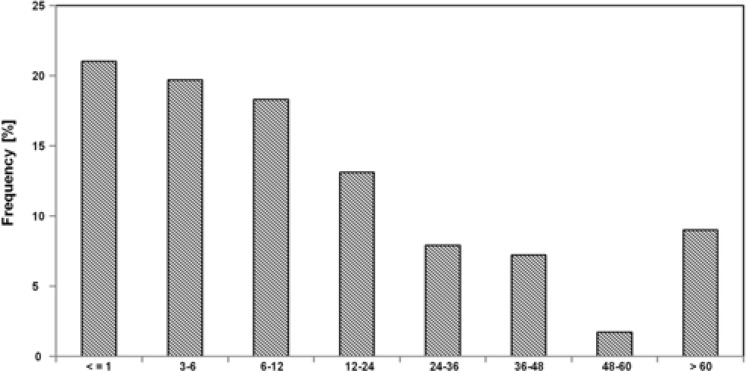
Distribution of last follow-up visits from less than 3 to more than 60 months post-surgery.

Data of last follow-up is available for 260 patients. Only 21% no longer attended after 3 months, a further 19.7% after 6 months and 18.3% after a year. Only 9% were seen after 5 years as indicated in Figure 2.

Pre-operatively, in 187 children, both eyes had been stated blind preoperatively, in 23 children 1 eye was blind and the other with severe visual impairment. There were 6 patients with 1 eye blind, the other eye severe visual impaired. In 59 children both eyes were visually severe impaired, in 9 children 1 eye was visually severe impaired and the other eye visual impaired. In 14 children both eyes were visually impaired.

Details of best corrected VA (BCVA) at last follow-up are given in tables 3 and 4.

**Table 3:** Influence of surgery on bilateral visual performance

	[%]	Hennig (2013) in %
**Improved**	81.9	94.1
**Stable**	16.2	-
**Deteriorated**	1.9	0.1

**Table 4:** Postoperative outcome of bilateral vision

	[%]	Hennig (2013) in %
**no visual impairment (6/18 or** **better), at least in 1 eye**	24.2	-
**(out of 24.1 % in both eyes**	(15.4)	-
**visual impairment (6/60 or** **better), at least in 1 eye**	40.4	-
**severe visual impairment** **(6/60–3/60), at least in 1 eye**	25.4	-
**Still blind in both eyes (< 3/60)**	10.0	5.6

BCVA at the last follow-up and its relation to preoperative BCVA is documented and our findings were compared with results published by Hennig and co-workers in their prospectively planned and well controlled study from 2013 8.

We found that 81.9% of patients had improved visual outcomes, 16.2% had no improvement and 1.9% had a worse visual outcome compared with the initial visual acuity (Tab. 3).

In detail, there was a good result (6/18 or better) in at least 1 eye found in 24.2% of children, out of these 15.4% had good vision in both eyes (Tab. 4).

There was a vison of 6/18 to 6/60 in at least in 1 eye in 40.4% and a vision of 6/60 to 3/60 in at least in 1 eye in 25.4%. 10.0% of the children remained blind in both eyes (worse than 3/60) (Tab. 4).

**Figure 3:**
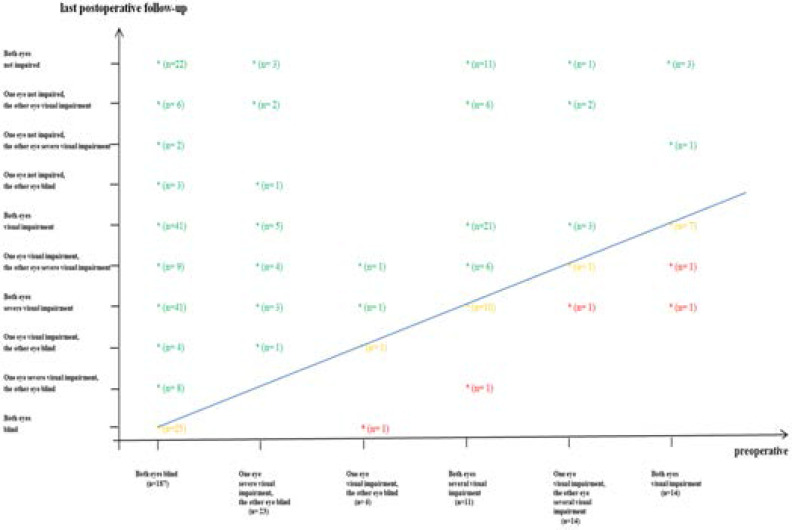
Best corrected visual acuity of bilateral paediatric cataract patients during last follow-ups. Green values indicate groups with increased BCVA; yellow values indicate groups with stable BCVA; red values indicate groups with decreased BCVA.

A more detailed view of surgical outcomes based on BCVA values of both eyes obtained during the last follow-ups in comparison with the pre-operative status is given in Figure 3.

## Discussion

According to WHO recommendations paediatric cataract surgery should be performed at tertiary care centres with trained personal, experienced in paediatric anaesthesia, fully equipped with automated vitrectomy machines, good objective refraction, and low vision services.

At the Eye Department of St. Joseph's Hospital there was extensive experience in minimal invasive cataract surgery for many years (up to 3000 patients annually). Ultrasound biometry and hand-held-keratometry as well as automated vitrectomy were introduced in 2002. These pre-requisites which had not been present at that time in any other eye department in Kinshasa, together with an enthusiastic group of volunteer social workers led to a rapid growth of the paediatric ophthalmic care unit. The team gradually enlarged their spectrum by incorporating strabismology, oculoplastic, and lacrimal surgery. The results have been published 9, 10. In a study published in 2007 which was carried-out in blind schools 10 out of 145 eyes were blind from cataract and the most common reason for blindness was found to be corneal opacification 11. Whether this reduction in cataract blind children from 33.7% (2000) to 6.9% (2007) was due to our initiative is difficult to decide as some other schools had been involved in the later survey.

Once a year visits from a team of the Rostock University Eye Department came for practical and theoretical support. It helped to develop the computer based documentation system applied for the preparation of this report. Regular discussions between the Rostock and the Kinshasa teams, and also in combination with international experts in the field concerning what age lens implantation surgery should be performed in children in developing countries led to conclusions that in places, where no contact lens supply is present and hardly any high quality aphakic spectacles correction can be guaranteed, it is perfectly justified to perform this kind of surgery.

The largest similar series have been published by Hennig and co-workers in a retrospective study 12. In 2011 there was a report of 2633 eyes with paediatric cataracts where only in 48 eyes no lens had been implanted for various reasons. A visual improvement could be achieved in 94.3%, nevertheless with a poor outcome (20/100 or worse) in 78.1% 12. In their next prospectively planned and well controlled study from 2013 the same group reported admirable good results after bilateral cataract surgery in 390 children 8. Their surgical experience is documented in teaching videos in the internet (https://www.youtube.com/watch?v=QzIVy8bcnag). Only 5.6% of children with more than 1 year follow-up were still blind. Our results compared in the Tables 3 and 4 are somewhere in between these two series and go in line with other case series from Shandong, China 13 and Gondar, Ethiopia 14 indicating that there is a good chance that a prospective study in Kinshasa (or a similar situation) could further improve the results in the near future.

Evidence from East Africa suggests the importance of subsidies for free cataract surgery to children, with a reimbursement of costs for spectacles, low vision services and transportation. It has been proven that this considerably helps to address the problem of childhood blindness 15–18. This is demonstrated by the fact that in 2011, where sponsor money was not available, surgical numbers dropped to 17 children during the year and significantly raised the years following, when a major amount was given by NGO's and reached its peak value with 44 in 2014.

The CBR program was involved in all steps prior to and after surgery, counselling the children and their parents. It was, in part, financed by charity NGO's who subsidised surgery costs in order to enable un-operated children with cataract, identified in the community, to have access to surgery and long-term follow-up services. The results have shown that this is an adequate way to clear up the backlog of paediatric cataract blindness in Kinshasa according to WHO recommendations 19, 20.

The median age at surgery in this series was 5.7 years which is similar to those reported in developing countries^21^–^23^. This late presentation is usual in Africa and was reported to be associated with transportation difficulties, lack of awareness and poverty 15. In this series most children were living in poor urban areas, with low family incomes. Poverty, lack of awareness of the disease among parents, prior consultation of traditional healers, and prayers for healing were facts that justified the late presentation for surgery in Kinshasa^24^. Increasing awareness of paediatric cataract and its management among healthcare personnel during their education, and among parents by the media, are crucial to increase access to eye care, including follow-up.

Boys presented more than girls for bilateral cataract surgery (64.9% to 35.1%). This concurs with other studies reported from developing countries 25, 26. In Kinshasa, there is no evident cultural, economic or religious trend to favour boys rather than girls to access medical care as stated in other studies 15–17. Nevertheless, studies must be carried out to elucidate why boys are apparently more often affected by cataract than girls in most of studies, and in many parts of the world 21, 26.

Out of the 298 patients data for last follow-ups are available for 260 children. The mean follow-up in this study was 2.8 ± 11.1 months (median 0.63 months) with a maximum of 122 months. The mean number of follow-up visits per patient was 4.22 ± 2.81 with the minimum of 1 visit and a maximum of 17 visits.

Transportation was not a significant barrier coming back for further visits as the CBR gave parents support for transportation. One of the reasons for the decrease of adherence to follow-up was that many parents were happy with the satisfactory functional vision of their children after surgery and did not find it necessary to return to the hospital for further improvement, or for help in preparing the child to be ready for school. Low vision services tried to offer incentives and to train the children in the utilization of devices where necessary, but most families did not accept them and seem to be happy with vision improvement as it was. Nevertheless, a long-term commitment to follow-up is needed for management of residual refractive errors and to identify and treat post-operative complications. Adequate follow-up is essential for the prevention of complications following childhood cataract surgery. Strategies have been recommended for the African population^27^. Restoration of sight from cataract blindness needs to be planned as a program with short term, medium, and long term goals, as support from the community needs to be sustained. Secondly, strategies for follow-up improvement such as cell phone reminder and reimbursement of transportation may help. Additionally, the involvement and role of a childhood blindness coordinator is essential and strongly recommendable 27.

The time between surgery and last follow-up varies from 3 months to 122 months. The information given in table 3 and 4 is based on the visual performance of the best eye at the last follow-up. Following this consideration, we have finally 24.2% of children with no visual impairment (15.4% no visual impairment in both eyes). This could be regarded as full visually rehabilitation, so these children should be able to attend normal schools without any constraint.

40.4% of children still have some visual impairment (6/60 or better) in the better eye, so they need special care and specialized optical corrections for schooling and any further education. This is even more relevant for 25.4% of children with a VA of 6/60 to 3/60 (severe visual impairment) at their last presentation. Reasons for vision lower then 6/18 are mostly the inability to perform proper amblyopia treatment under the given circumstances. For future studies VA of 6/18 as an equivalent for the ability to go to a normal school should be included in the electronic data base.

Comparing pre- and post-operative BCVA (at the last visit) there is an improvement of visual performance in 81.9% of the children, 16.2% remained unchanged and in 1.9% deterioration was found (Tab. 4, Fig. 3). Detailed analysis of these results is difficult as documentation needs to be improved for future analysis.

Comparing our results with other publications it is mainly the group from Lahan that reported on bilateral cataract surgery with lens implantation in children 8, 12. In their first retrospective study published in 2011, 2003 eyes (7.7% monolateral) they achieved an improvement in 94% of eyes with only 0.1% of deterioration. Our results could certainly be improved with a prospective study, comparable with Hennig's study about 390 children with bilateral cataract from Nepal and Northern India 8.

The situation in Kinshasa differs in many aspects, but by learning from these first results, the ongoing prospective study will most likely further improve the surgical outcome. We are hoping that the social situation in the poor areas of the town, where most of the patients came from, will improve too.

Complications that also negatively inluence outcomes can be divided into short-term and long-term. Short-term complications (2.3%) include retinal detachment and IOL decentration. Long-term complications (7.3%) are composed of the groups of secondary glaucoma and secondary cataracts. The complication rates we have observed seem very low compared to other case series 20, 28. However, it should be noted that in our study only bilaterally operated cataract patients were examined postoperatively. When we started the program we performed surgery in bilateral cases sequentially. Having noticed that there was hardly any surgery related problem in these children we decided to operate both eyes simultaneously to 1) reduce the risk of a second general anaesthesia and 2) to avoid the behaviour of non-representation of families for the second eye. When complications occur in only one eye, patients /parents often remain satisfied with the surgical outcome and do not always see the need for further examination / follow-up under the given circumstances. These patients thus escape detection. In order to avoid the phenomenon that the worse eye is not being seen by the ophthalmologist under the given circumstances, the role of a childhood blindness coordinator should not be underestimated as stated by Kishiki et al. 27.

## Conclusion

At the last follow-up of children with bilateral cataract following the data of figure 3 and looking at the change of visual performance of each single individual the following statements can be made.

Post-operative visual outcome of vision under bilateral considerations:

Summarising the influence of surgery on bilateral visual performance revealed that we improved vision in 213 children (81.9%) (Hennig, 2011 in 94.1%). In 42 (16.2%) vision remained unchanged and in 5 (1.9%) vision deteriorated (Hennig 0.1%).

It is recommended that the incorporation of routine cataract surgical outcome auditing should be performed per surgical theatre session, per surgeon, and per centre. This has been seen to facilitate consist improvement in surgeons and centre surgical outcomes and enhances a reduction in surgical complication over time. Also it assists in the facilitation of individual surgeon's selfdevelopment in cataract surgery. Furthermore, a prospective survey with greater emphasis on early identification, good surgical follow-up and sensitisation of the community (and in particular the parents) of the importance of adequate follow-up through the CBR program should improve results.
